# Ipsilateral Recurrence of DCIS in Relation to Radiomics Features on Contrast Enhanced Breast MRI

**DOI:** 10.3390/tomography8020049

**Published:** 2022-03-01

**Authors:** Ga Eun Park, Sung Hun Kim, Eun Byul Lee, Yoonho Nam, Wonmo Sung

**Affiliations:** 1 Department of Radiology, Seoul St. Mary’s Hospital, College of Medicine, The Catholic University of Korea, Seoul 06591, Korea; hoonhoony@naver.com (G.E.P.); silver4095@naver.com (E.B.L.); 2 Division of Biomedical Engineering, Hankuk University of Foreign Studies, Yongin 17035, Korea; yhnam83@gmail.com; 3 Department of Biomedical Engineering, Research Institute of Biomedical Engineering, College of Medicine, The Catholic University of Korea, Seoul 06591, Korea; wsung@catholic.ac.kr

**Keywords:** breast cancer, quantitative imaging, magnetic resonance imaging

## Abstract

The purpose of this retrospective study was to investigate the association between ipsilateral recurrence of ductal carcinoma in situ (DCIS) and radiomics features from DCIS and contralateral normal breast on contrast enhanced breast MR imaging. A total of 163 patients with DCIS who underwent preoperative MR imaging between January 2010 and December 2014 were included (training cohort; *n* = 117, validation cohort; *n* = 46). Radiomics features were extracted from whole tumor volume of DCIS on early dynamic T1-subtraction images and from the contralateral normal breast on precontrast T1 and early dynamic T1-subtraction images. After feature selection, a Rad-score was established by LASSO Cox regression model. Performance of Rad-score was evaluated by the receiver operating characteristic (ROC) curve and Kaplan Meier curve with log rank test. The Rad-score was significantly associated with ipsilateral recurrence free survival (RFS). The low-risk group with a low Rad-score showed higher ipsilateral RFS than the high-risk group with a high Rad-score in both training and validation cohorts (*p* < 0.01). The Rad-score based on radiomics features from DCIS and contralateral normal breast on breast MR imaging showed the potential for prediction of ipsilateral RFS of DCIS.

## 1. Introduction

The incidence of ductal carcinoma in situ (DCIS) has increased significantly with the broad adoption of mammography screening, from 1–2% to nearly 20% of newly developed breast cancer in about 30 years [1,2]. DCIS is a non-invasive disease with a high probability of long-term, disease-free survival but also it has a highly heterogeneous disease course [3]. In the absence of appropriate radiation or endocrine therapy after breast conserving surgery (BCS), ipsilateral recurrence is observed in about 10–30% of patients [4,5]. There have been results that such adjuvant therapy reduces the risk of local recurrence by 30–50% [6,7,8,9]. Recently, several studies have been conducted to classify the risk of DCIS to determine treatment strategy and prognosis. The goal of these studies was to reduce overtreatment in the low-risk group [10] and to reduce the risk in the high-risk group, where the risk of invasive recurrence is up to 50% [7].

Radiomics analysis is a statistical method to analyze the surface characteristics and to identify and recognize an object. This characterization is based on the spatial distribution, signal intensity, and gray level co-occurrence of the images [11,12]. It has been applied to breast imaging to differentiate between benign and malignant lesions [13] to predict pathologic or prognostic factors [14,15], and to identify the response association for neoadjuvant chemotherapy [16,17]. There are recent radiomics studies focused on DCIS. Two studies attempted to predict upstaging of DCIS using preoperative mammography [18,19]. One study was about risk stratification of DCIS using preoperative contrast-enhanced MR [20].

The purpose of our study was to evaluate radiomics features from DCIS and contralateral normal breast composition on preoperative breast MRI as a prognostic factor for predicting ipsilateral recurrence of DCIS.

## 2. Materials and Methods

### 2.1. Study Population

**Figure 1:**
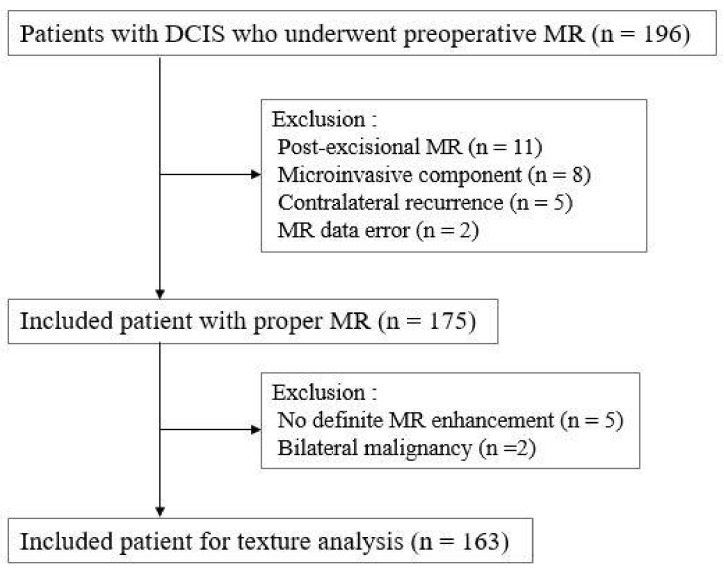
Flow chart for study population and exclusion criteria.

A total of 196 consecutive women who underwent preoperative breast MR imaging were diagnosed with DCIS between January 2010 and December 2014 in our institution. We excluded patients with post-excisional MR (*n* = 11), micro-invasive component in final pathology (*n* = 8), contralateral recurrence (*n* = 5) and MR data error (*n* = 2). In addition, we excluded patients with no definite enhancement on MR exam (*n* = 5) and bilateral malignancy (*n* = 2). Finally, 163 consecutive women (median age, 52.5 years; range, 31–77 years) were included in this study (Figure 1).

### 2.2. Radiomics Feature Extraction

**Figure 2:**
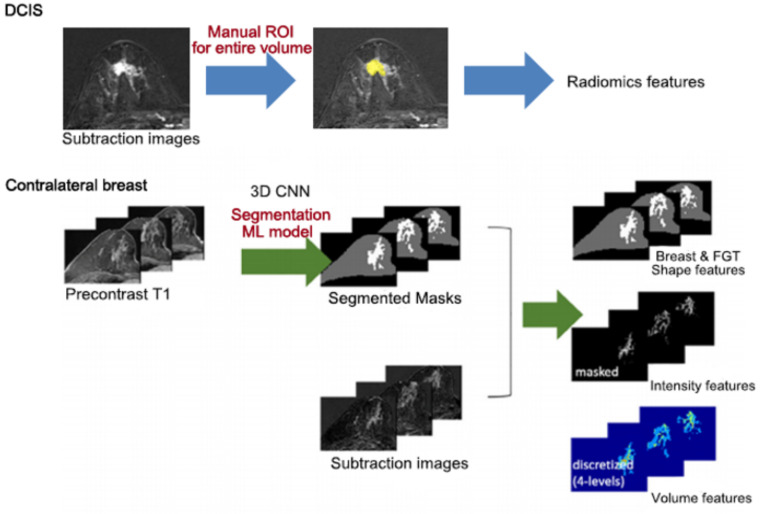
Overall process of radiomics features extraction.

Figure 2 shows overall process of radiomics analysis of DCIS and contralateral normal breast. For the DCIS lesion, we used post-processing software (Olea Sphere, Version 3.0, Olea Medical, La Ciotat, France) for semiautomatic segmentation and feature extraction. The entire tumor volume was segmented on axial T1-weighted early dynamic contrast subtraction images derived from the PACS system. The index tumor and tumor boundary were delineated by three radiologists (K.S.H, P.G.E. and L.E.B, with 20 years, 4 years of experience in breast MR imaging and a senior radiology resident, respectively) by consensus. The segmented volume was used as a mask for the extraction for 108 features of seven categories (Supplementary Table S1).

For contralateral breast composition we used a previously developed machine learning based fully automatic segmentation and classification model based on a 3D convoluted neural network (CNN) [21]. First, this model used precontrast axial T1-weighted images to make segmentation masks of the whole breast and fibroglandular tissue (FGT). The potential background parenchymal enhancement (BPE) voxels were estimated from an early T1 subtraction image using a segmented FGT mask. During the process, we extracted a total of 62 radiomics features from each whole breast mask and fibroglandular tissue mask in contralateral normal breast (Supplementary Table S2). The automatic classification model for FGT grade and BPE level based on BI-RADS 5th edition lexicon was implemented using the ensemble tree model from the predefined radiomics features [22].

### 2.3. Clinico-Pathological Analysis

Medical records and pathologic reports from surgical excision and core needle biopsy were reviewed: surgical type, DCIS size, nuclear grade, comedo-type necrosis, hormone receptor and resection margin status, radiation and endocrine therapy, and last outpatient follow-up. In this study, last outpatient follow-up was defined as the duration from surgery to the last outpatient visit or ipsilateral recurrence. DCIS lesions were classified according to Van Nuys Pathologic Grade (VNPG) and COMET (Comparison of Operative versus Monitoring and Endocrine Therapy) classification. The VNPG is based on nuclear grade and necrosis; grade 1 as non-high nuclear grade without comedo-type necrosis, grade 2 as non-high nuclear grade with comedo-type necrosis, and grade 3 as high nuclear grade with or without comedo-type necrosis [23]. In this study, we considered low risk as VNPG grade 1 and non-low risk as VNPG grade 2 or grade 3. The COMET classification was also applied. The low-risk group was defined as non-high nuclear grade of DCIS lesions, estrogen receptor (ER) positive with or without progesterone receptor (PR) positive, and human epidermal growth factor receptor 2 (HER2) scores 0, 1+, or 2+ by immunohistochemistry. The non-low risk group was defined as high-nuclear grade of DCIS, ER negative, and HER2 score 3+ by immunohistochemistry [24].

### 2.4. MR Imaging Acquisition

All breast MR examinations were performed in the prone position using a dedicated eight-channel breast surface coil from two different vendors (3-T Verio; Siemens Healthcare, Erlangen, Germany; 1.5-T Signa; GE Medical Systems, Milwaukee, WI, USA). Images were obtained using the following sequences: (1) axial turbo spin-echo T2-weighted imaging (T2WI); (2) axial diffusion-weighted imaging (DWI) with two sequences and automatically calculated apparent diffusion coefficient (ADC) maps; (3) pre-contrast and post-contrast, fat-suppressed axial T1-weighted imaging(T1W1) obtained before and five different times after the rapid bolus injection of gadolinium DTPA (Gd-DTPA, 0.1 mmol/kg Gadovist; Bayer Schering Pharma, Berlin, Germany). For 3-T Verio, axial T1-weighted flash three-dimensional volumetric interpolated brain examination (VIBE) sequences were obtained with a TR/TE of 4.4/1.7, a flip angle of 10°, a slice thickness of 1.2 mm, and an acquisition time of 1 min. The images were obtained before and at a 10, 70, 130, 190, 250 and 310 s after an injection of contrast agent. For 1.5-T Signa, axial spin-echo T1WI was with a TR/TE of 6.2/3.1, a flip angle of 10°, a slice thickness of 2.6 mm, and an acquisition time of 1 min 31 s. The images were obtained before and at a 91, 192, 273, 364 and 455 s after an injection of contrast agent.

### 2.5. Statistical Analysis

The continuous variables were presented as mean ± standard deviation or median and quartile. The categorical variables were presented as frequencies and percentage. Mann-Whitney U or the Wilcoxon rank sum test was used to compare the baseline characteristics for continuous variables, and the Chi-square test or Fisher’s exact test for categorical variables.

Univariate Cox Proportional hazard regression and then LASSO Cox regression was used to narrow down significant radiomics features associated with ipsilateral recurrence of DCIS. The “Rad-score” and “Rad-score combined with clinical feature” were calculated based on selected radiomics features (Supplementary Files S3 and S4). Each model was evaluated and compared using the area under the receiver operating characteristic (ROC) curve. Then, we divided the high-risk group and low-risk groups using cutoff values according to the Youden index. The Kaplan-Meier curve with Log rank test were used to comparison of recurrence free survival between training set and validation set, and between the high-risk group and low-risk group. Statistical analysis was performed using a commercial software (SPSS, Version 19.0; Chicago, IL, USA) and R version 2.15.3 (R Foundation, Vienna, Austria). Statistical significance was defined as *p* < 0.05.

## 3. Results

### 3.1. Baseline Patients Characteristics

Table 1 shows baseline characteristics between patients with ipsilateral recurrence and patients with no recurrence. Ipsilateral recurrence occurred in 10 patients (6%, 10 of 163) at a median of 51.5 months (range 12–113 months). COMET classification and increased BPE level in preoperative MR were associated with ipsilateral recurrence of DCIS (*p* < 0.05). Age showed marginal significance (*p* = 0.05). Among the patients with ipsilateral recurrence, half of patients (five of ten) developed invasive recurrent cancer, and the other half developed DCIS. Eighty percent of the recurrent cancers (eight of ten) occurred in patients who had high-grade DCIS. Ninety percent of the patients (nine of ten) underwent BCS. No patients developed distant metastasis or breast cancer-related death.

### 3.2. Feature Selection and Rad-Score Calculation

A total of 163 patients were included with 117 patients in the training cohort and 46 patients in the validation cohort, using stratified random sampling. There were no significant differences in recurrence and clinical features between the training set and validation set. (Table 2). A total of 20 radiomics features were significant in Univariate Cox proportional regression in training cohort. We narrowed down to five features using LASSO Cox regression. The Rad-score was established via linear combination of the selected five features multiplied by their respective LASSO Cox coefficients (Supplementary File S3).


$$\mathrm{Rad}\;\mathrm{score}=0.2974\times Total\;Energy\left(DCIS\right)+0.2988\times Entropy\left(breast\;mask\right)\;\;+0.5835\times 75th\;percentile\left(breast\;mask\right)\;\;+0.3105\times interquartile\;range\left(FGT\;mask\right)\;\;+0.0665\times volume\;threshold\left(FGT\;mask\right)$$

Two models that applied clinical features to Rad-score were also calculated through a similar process to the above (Supplementary File S4).

### 3.3. Rad-Score Assessment

**Figure 3:**
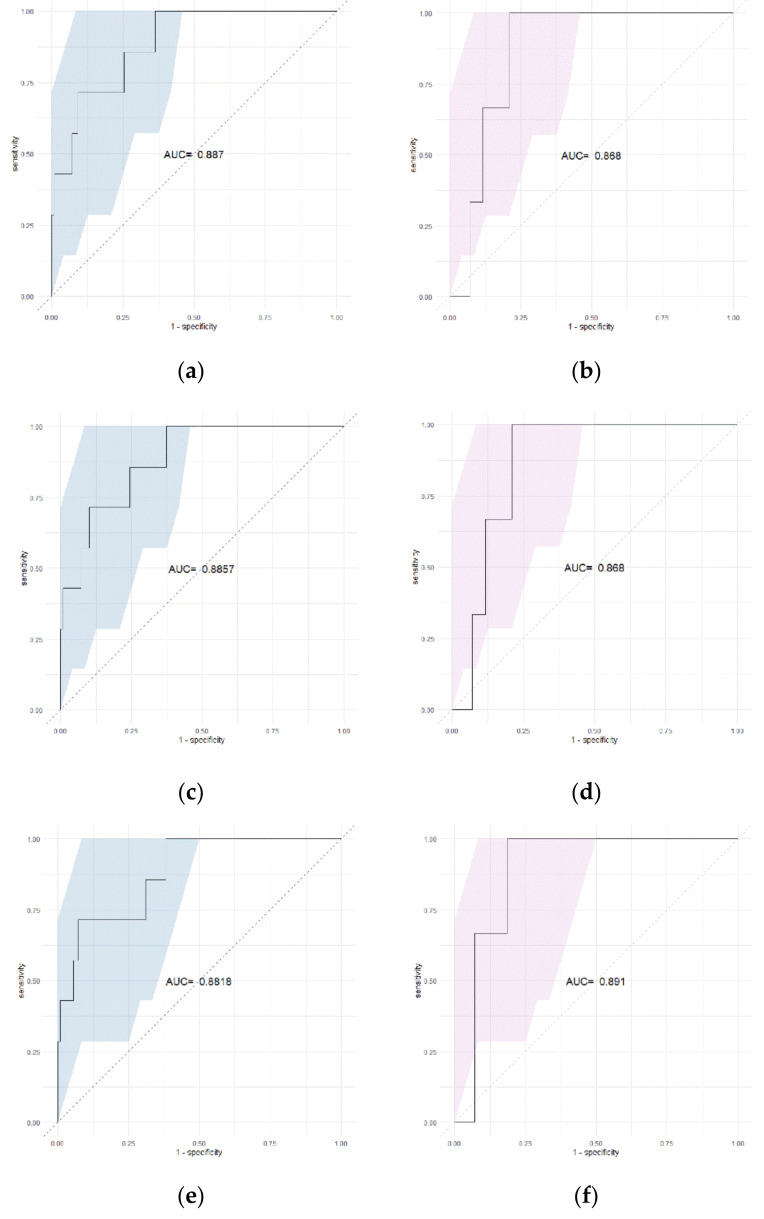
ROC curve of (**a**) Rad-score (training cohort), (**b**) Rad-score (validation cohort), (**c**) Rad-score + age (training cohort), (**d**) Rad-score + age (validation cohort), **(e**) Rad-score + COMET classification (training cohort), (**f**) Rad-score + COMET classification (validation cohort) * colored area = 95% CI (confidence interval).

The Rad-score was effective in predicting ipsilateral recurrence in the training cohort (AUC 0.887, 95% CI 0.7765–0.9975) and validation cohort (AUC 0.868, 95% CI 0.7495–0.9869). In the Rad-score + age model, AUC of 0.8857 (95% CI 0.7744–0.997) in the training cohort and 0.868 (95% CI 0.7495–0.9869) in the validation cohort. In the comparison of ROC curves, these three models showed no significant difference in both the training cohort and validation cohort (*p* > 0.05). In the Rad-score + COMET classification model, AUC was 0.8818 (95% CI 0.7596–1) in the training cohort and 0.891 (95% CI 0.7808–1) in the validation cohort (Figure 3). In a comparison of ROC curves, these three models showed no significant difference in both the training cohort and validation cohort (*p* > 0.05).

**Figure 4:**
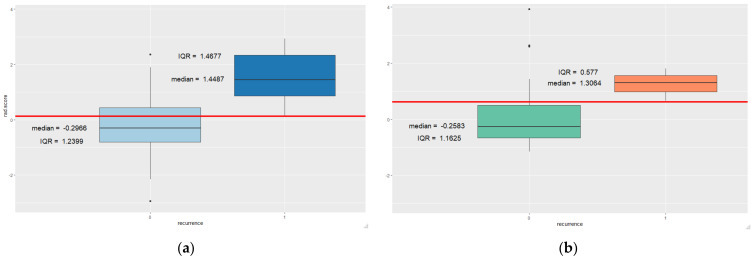
Distribution of the Rad-score according to ipsilateral recurrence in training (**a**) and validation (**b**) cohorts red line = cut off value, 0 = no recurrence, 1 = recurrence group.

Figure 4 shows the distribution of Rad-scores in the recurrence and non-recurrence groups. In the training cohort, the Rad-score was significantly higher in the group with ipsilateral recurrence (median, 1.4487; interquartile range, 1.4677) than the group without recurrence (median, −0.2966; interquartile range, 1.2399). In the validation cohort, the Rad-score was significantly higher in the group with ipsilateral recurrence (median, 1.3064; interquartile range, 0.577) than the group without recurrence (median, −0.2583; interquartile range, 1.1625). Cut-off values from ROC curve were 0.126 and 0.618, respectively.

**Figure 5:**
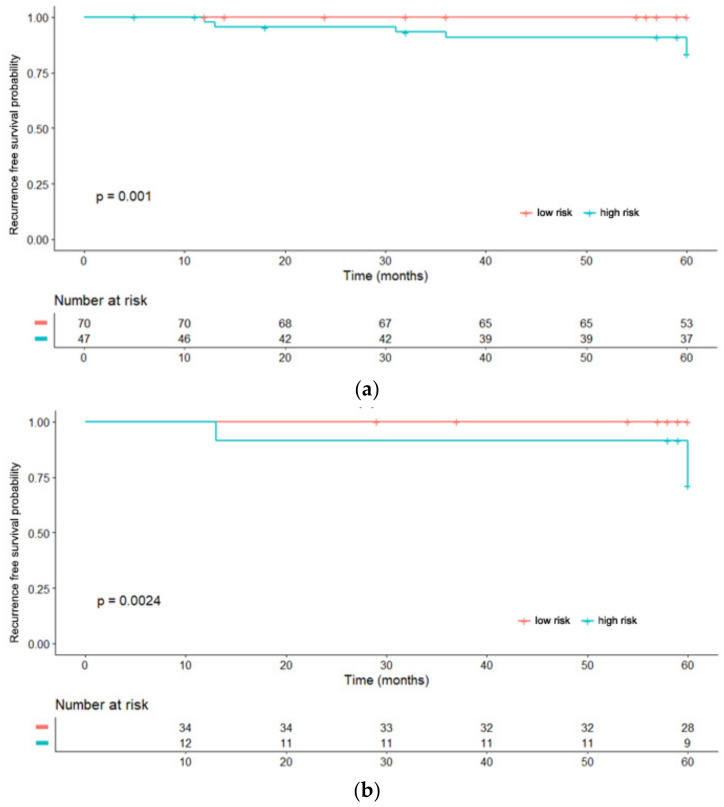
Kaplan-Meier analysis according to the risk groups in training (**a**) and validation (**b**) cohorts.

We further divided patients into low-risk and high-risk groups based on obtained cut off values and performed Kaplan-Meier analysis to validate the prognostic value of Rad-score (Figure 5). The Rad-score was significantly associated with ipsilateral recurrence free survival (RFS). The low-risk group with a low Rad-score showed higher ipsilateral RFS than the high-risk group with a high Rad-score in both training and validation cohorts (*p* < 0.01).

## 4. Discussion

This study assessed radiomics features of DCIS and contralateral normal breast composition on preoperative breast MRI as prognostic factors for predicting ipsilateral recurrence of DCIS. Several studies have been conducted on radiomics analysis for evaluation of invasive cancer and recurrence [25,26]. To the best of our knowledge, this is the first attempt to predict ipsilateral recurrence of DCIS using radiomics features from both DCIS lesion and contralateral normal breast. The Rad-score in our study was capable of stratifying patients into low and high risk of recurrence and was significantly higher in patients with ipsilateral recurrence. The Rad-score represented no significant difference in performance when compared to the model combining Rad-score with clinical feature.

The full mechanism behind the relationship between radiomics features and recurrence has not been elucidated. A previous study found that radiomics features were closely related to tumor biology and microscopic structures [27]. Among the selected features, Total Energy from DCIS lesion was included in the Rad-score. Total Energy is the value of Energy feature scaled by the volume of the voxel in cubic mm. Most features of Rad-score were selected from the intensity and volume-based features from T1-weighted subtraction images of contralateral breast. The 75th percentile of voxel intensities of segmented breast mask in contralateral breast showed the highest coefficient value. Prior studies analyzed DCIS recurrence using dynamic contrast-enhanced (DCE) MR imaging. Kim et al. [28] showed that parenchymal signal enhancement ratio (SER) and tumor size were associated with ipsilateral recurrence after breast conserving surgery in DCIS patients. Luo et al. [29] proved that mean BPE, functional tumor volume, and peak SER were associated with recurrence in a case-control study. BPE, which is the enhancement of normal fibroglandular tissue on contrast enhanced dynamic breast MR, is related to the vascular microenvironment and glandular concentration in histopathologic study [30]. Increased BPE is also associated with increased metabolic activity, which could potentially provide more favorable environment for tumor growth [31]. Although there are differences in study design, several studies have demonstrated that BPE is associated with developing risk of breast cancer, including DCIS [32,33,34,35].

Our derived model could be interpreted to suggest the underlying breast environment may have contributed more to the ipsilateral recurrence than the tumor biology of DCIS itself. However, the value of radiomics features obtained from contralateral breast is still unclear, and further studies with a larger number of cases are required.

There are limitations in this study. First, it was a retrospective study from a single institution. Second, the number of participants was small, especially the number of ipsilateral recurrence events. An inherent limitation was the noninvasive nature of DCIS. Third, we only analyzed the early dynamic phase of the DCE T1-weighted images, since DCE T1 is the most important phase in breast cancer evaluation. Third, the radiomics features for DCIS were obtained via manual segmentation. Although MR is the most sensitive modality for identifying DCIS, DCIS is commonly manifested as a non-mass enhancement with various enhancement patterns [31]. Therefore, manual segmentation of DCIS was inevitable. For this reason, the boundary was somewhat subjective in the process of drawing the ROI to encompass the whole tumor volume. We tried to reduce errors through consensus between radiologists.

## 5. Conclusions

In conclusion, the Rad-score based on MR imaging feature demonstrated the potential for predicting ipsilateral recurrence in DCIS patients. The radiomics features extracted from both the DCIS lesion and the contralateral normal breast were significant in recurrence prediction. These findings may be helpful for risk stratification and for personalized treatment.

## Figures and Tables

**Table 1:** Baseline characteristics in the recurrence and non-recurrence.

	Patients with No Recurrence (*n* = 153)	Patients with Ipsilateral Recurrence (*n* = 10)	*p*-Value
Age	52.9 ± 9.8	46.5 ± 7.5	0.05
Last outpatient follow-up (months)	82.2 ± 26.5	51.7 ± 36.1	0.009
Surgery type			0.109
Total mastectomy	100	9	
BCS	53	1	
Radiation therapy			0.343
No	100	8	
Yes	53	2	
Endocrine therapy			0.607
No	111	8	
Yes	42	2	
DCIS size	2.51 ± 1.89	2.35 ± 1.99	0.814
DCIS nuclear grade			0.075
Non-high	75	2	
High	78	8	
Comedo necrosis			0.128
Negative	50	1	
Positive	101	9	
ER			0.078
Negative	115	7	
Positive	38	3	
PR			0.765
Positive	100	7	
Negative	53	3	
HER2			0.479
Negative	108	6	
Positive	45	4	
Ki-67 (%)			0.669
<14%	94	5	
≥14%	56	4	
IHC type			0.562
Luminal	114	7	
HER2-enriched	26	3	
Basal-like	12	0	
VNPG			
Low risk	38	1	0.287
Non-low risk	115	9	
Comet classification			0.031
Low risk	68	1	
Non-low risk	84	9	
Resection margin (2 mm)			0.078
Negative	136	7	
Positive	17	3	
Fibroglandular tissue (FGT)			0.743
Almost entirely fat, Scattered	54	3	
Heterogenoues, Extreme	99	7	
Background parenchymal enhancement (BPE)			0.028
Minimal, Mild	122	5	
Mild, Marked	31	5	

Results are presented as number (percentage) for categorical variables and mean (SD) for continuous variables. *p* values were calculated using Chi-square test or Fisher’s exact test for categorical variables and Wilcoxon rank sum test for continuous variables.

**Table 2:** Patient characteristics in the training and validation cohorts.

	Training Cohort (*n* = 117)	Validation Cohort (*n* = 46)	*p*-Value
Age	53.5 ± 10.2	50.1 ± 8.13	0.12
Surgery type			0.41
Total mastectomy	42	12	
BCS	75	34	
Radiation therapy			0.41
No	44	11	
Yes	73	35	
Endocrine therapy			0.10
No	28	12	
Yes	78	41	
Ipsilateral recurrence			1 *
No recurrence	110	43	
Recurrence	7	3	
DCIS size (cm)	2.52 ± 1.88	2.43 ± 1.94	0.62
DCIS nuclear grade			0.34
Non-high	58	19	
High	59	27	
Comedo necrosis			0.17
Negative	41	11	
Positive	76	35	
ER			0.80
Negative	29	12	
Positive	88	34	
PR			0.86
Negative	34	20	
Positive	72	33	
HER2			0.23
Negative	85	29	
Positive	32	17	
Ki-67 (%)	14.8 ± 14.7	10.9 ± 8.21	0.41
IHC_type			0.69
Luminal	88	34	
HER2-enriched	19	10	
Basal-like	10	2	
VNPG			0.22
Low risk	31	8	
Non-low risk	86	38	
COMET classification			0.33
Low risk	53	17	
Non-low risk	64	29	
Resection margin (2 mm)			0.85
Negative	103	40	
Positive	14	6	
Fibroglandular tissue (FGT)			0.79
Almost entirely fat, Scattered	43	14	
Heterogeneous, Extreme	74	32	
Background parenchymal enhancement (BPE)			0.70
Minimal, Mild	93	34	
Moderate, Marked	24	12	

Results are presented as number (percentage) for categorical variables and mean (SD) for continuous variables. *p* values were calculated using Pearson’s Chi-square test or Fisher’s exact test * for categorical variables and Wilcoxon rank sum test for continuous variables.

## Data Availability

All data generated and analyzed during this study are included in this published article. Raw data supporting the findings of this study are available from the corresponding author on request.

## Supplementary Materials

The following supporting information can be downloaded at: https://www.mdpi.com/article/10.3390/tomography8020049/s1. Table S1: Entire volume based extracted radiomics features from DCIS lesion. Table S2: Extracted radiomics features from breast mask and fibroglandular tissue mask of contralateral normal breast. File S3: Feature selection and Rad-score calculation. File S4: Rad-score combined with clinical feature.
