# Supplementary material for: Ipsilateral Recurrence of DCIS in Relation to Radiomics Features on Contrast Enhanced Breast MRI
Source: Tomography. 2022 Mar 1;8(2):596–606. doi: 10.3390/tomography8020049 (PMC8938812; doi:10.3390/tomography8020049)
Supplement: Supplementary file 1 [file tomography-08-00049-s001.zip › tomography-1554300-supplementary.pdf]

Supplementary Table S1. Entire volume based extracted radiomics features from DCIS lesion

Category	Parameters
First order	Energy, Total Energy, Entropy, Minimum, 10th Percentile, 90th Percentile, Maximum, Mean, Median, Interquartile Range, Range, Mean Absolute Deviation, Robust Mean Absolute Deviation, Root Mean Squared, Standard Deviation, Skewness, Kurtosis, Variance, Uniformity
Shape	Volume, Surface Area, Surface Area to Volume Ratio, Sphericity, Compactness 1, Compactness 2, Spherical Disproportion, Maximum 3D Diameter, Maximum 2D Diameter Slice, Maximum 2D Diameter Column, Maximum 2D Diameter Row, Major Axis, Minor Axis, Least Axis, Elongation, Flatness
Gray Level Co-occurrence Matrix (GLCM)	Autocorrelation, Joint Average, Cluster Prominence, Cluster Shade, Cluster Tendency, Contrast, Correlation, Difference Average, Difference Entropy, Difference Variance, Joint Energy, Joint Entropy, Informal measure of correlation 1, Informal measure of correlation 2, Inverse Difference Moments, Inverse Difference Moment Normalized, Inverse Variance, Maximum Probability, Sum Average, Sum Entropy, Sum of Squares
Gray Level Run Length Matrix (GLRLM)	Short Run Emphasis, Long Run Emphasis, Gray Level Non Uniformity, Gray Level Non Uniformity Normalized, Run Length Non Uniformity, Run Length Non Uniformity Normalized, Run Percentage, Gray Level Variance, Run Variance, Run Entropy, Low Gray Level Run Emphasis, High Gray Level Run Emphasis, Short Run Low Gray Level Emphasis, Short Run High Gray Level Emphasis, Long Run Low Gray Level Emphasis, Long Run High Gray Level Emphasis
Gray Level Size Zone Matrix (GLSZM)	Small Area Emphasis, Large Area Emphasis, Gray Level Non Uniformity , Gray Level Non Uniformity Normalized, Size Zone Non Uniformity, Size Zone Non Uniformity Normalized, Zone Percentage, Zone Entropy, Low Gray Level Zone Emphasis, Small Area Low Gray Level Emphasis, Small Area High Gray Level Emphasis, Large Area Low Gray Level Emphasis, Large Area High Gray Level Emphasis
Neighboring Gray	Coarseness, Contrast, Busyness, Complexity, Strength

Tone Difference Matrix	
Gray Level Dependence Matrix (GLDM)	Small Dependence Emphasis, Large Dependence Emphasis, Gray Level Non Uniformity, Dependence Non Uniformity, Dependence Non Uniformity Normalized, Gray Level Variance, Dependence Variance, Dependence Entropy, Low Gray Level Emphasis, High Gray Level Emphasis, Small Dependence Low Gray Level Emphasis, Small Dependence High Gray Level Emphasis, Large Dependence Low Gray Level Emphasis, Large Dependence High Gray Level Emphasis

Supplementary Table S2. Extracted radiomics features from breast mask and fibroglandular tissue mask of contralateral normal breast

Category	Parameters
Intensity	Energy, Entropy, 10th Percentile, 25th Percentile, 75th Percentile, 90th Percentile, Mean, Median, Interquartile Range, Range, Mean Absolute Difference, Root Mean Squared, Standard Deviation, Skewness, Kurtosis, Variance
Shape	Volume, Surface Area, Solidity, Equivalent Diameter, Extent, Convex volume, Principal Axis Length 0, Principal Axis Length 1, Principal Axis Length 2, Orientation 0, Orientation 1, Orientation 2
Volume	Volume threshold 1, Volume threshold 2, Volume threshold 3, Volume threshold 4

Intensity and volume based features were extracted from axial T1 early phase subtraction images

Shape based features were from axial T1-weighted images

Supplementary File S3. Feature selection and Rad-score calculation

Among 108 radiomics features from DCIS, 62 radiomics features from contralateral breast (breast mask and FGT mask), total 20 radiomics features (6 features from DCIS and 14 features from contralateral breast) were significant in Univariate Cox proportional regression in training

cohort.

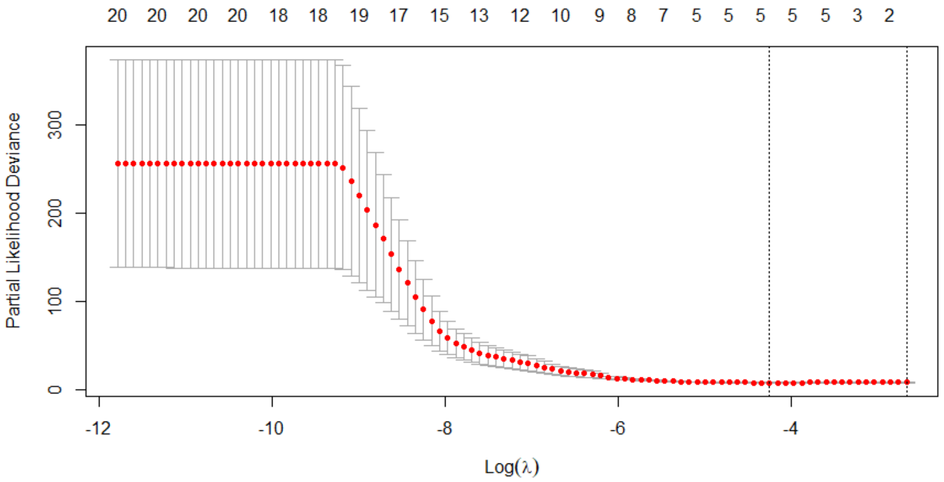

(a)

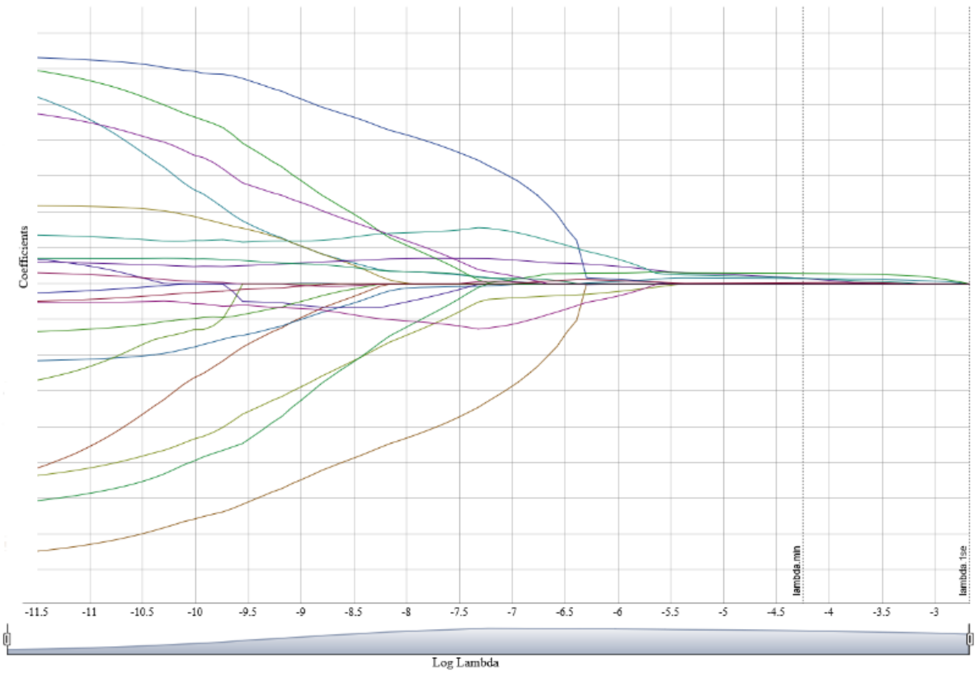

(b)

File S3-1. Feature selection suing the least absolute shrinkage and selection operator (LASSO) regression. (a) The partial likelihood variance was plotted versus log (λ). The turning parameter (λ = -4.247) was selected in the LASSO model. (b) LASSO coefficient profiles of 20 radiomics features was plotted versus log (λ). Total five radiomics features with non-zero

coefficients at λ = -4.247 were selected to build the Rad-score.

File S3-2. Selected radiomics features and LASSO coefficient

Radiomics features	LASSO coefficient
Total Energy (from DCIS)	0.2974
Entropy (from breast mask, contralateral breast)	0.2988
75th percentile (from breast mask, contralateral breast)	0.5835
Interquartile range (from FGT mask, contralateral breast)	0.3105
Volume threshold 2 (from FGT mask, contralateral breast)	0.0665

The Rad-score was established via linear combination of the selected five features multiplied by their respective LASSO Cox coefficients.

Rad-score = 0.2974 x Total Energy (DCIS) + 0.2988 x Entropy (breast mask) + 0.5835 x 75th percentile (breast mask) + 0.3105 x interquartile range (FGT mask) + 0.0665 x volume threshold 2 (FGT mask)

Supplementary File S4. Rad-score combined with clinical feature

Rad-score combined with clinical feature was calculated through the following process.

First, we selected “age” and “COMET classification” among clinical features. That is because two clinical features showed significant difference between recurrence group and non-recurrence group. We used 20 radiomics features that were significant in Univariate Cox regression. Then, LASSO Cox regression was performed on a total 21 features (20 radiomics feature + 1 clinical feature). The “Rad-score + clinical feature” equation was constructed by applying the LASSO coefficients to the selected features (same method as in Supplementary

File S3).

Rad-score + Age = 0.2923 x Total Energy (DCIS) + 0.2724 x Entropy (breast mask) + 0.5749 x 75th percentile (breast mask) + 0.2856 x interquartile range (FGT mask) + 0.0657 x volume threshold 2 (FGT mask) – 0.0040 x age

Rad-score + COMET classification = 0.2886 x Total Energy (DCIS) + 0.2877 x Entropy (breast mask) + 0.5598 x 75th percentile (breast mask) + 0.3733 x interquartile range (FGT mask) + 00252 x volume threshold 2 (FGT mask) + 0.2304 x COMET classification
